# Spontaneous Quaternary and Tertiary T-R Transitions of Human Hemoglobin in Molecular Dynamics Simulation

**DOI:** 10.1371/journal.pcbi.1000774

**Published:** 2010-05-06

**Authors:** Jochen S. Hub, Marcus B. Kubitzki, Bert L. de Groot

**Affiliations:** 1 Department of Cell and Molecular Biology, Uppsala University, Uppsala, Sweden; 2 Computational Biomolecular Dynamics Group, Max-Planck-Institute for Biophysical Chemistry, Göttingen, Germany; University of Tokyo, Japan

## Abstract

We present molecular dynamics simulations of unliganded human hemoglobin (Hb) A under physiological conditions, starting from the **R**, **R2**, and **T** state. The simulations were carried out with protonated and deprotonated HC3 histidines His(β)146, and they sum up to a total length of 5.6µs. We observe spontaneous and reproducible **T**→**R** quaternary transitions of the Hb tetramer and tertiary transitions of the α and β subunits, as detected from principal component projections, from an RMSD measure, and from rigid body rotation analysis. The simulations reveal a marked asymmetry between the α and β subunits. Using the mutual information as correlation measure, we find that the β subunits are substantially more strongly linked to the quaternary transition than the α subunits. In addition, the tertiary populations of the α and β subunits differ substantially, with the β subunits showing a tendency towards **R**, and the α subunits showing a tendency towards **T**. Based on the simulation results, we present a transition pathway for coupled quaternary and tertiary transitions between the **R** and **T** conformations of Hb.

## Author Summary

As the prototypic allosteric protein, human hemoglobin (Hb) has drawn extensive scientific efforts for many decades. Human Hb exists in two quaternary conformations, the low-affinity (or deoxy) **T** state, and the high-affinity (or oxy) **R** state, and the transition between the **T** and the **R** state is mainly characterized by a 15° rotation of the α1β1 dimer with respect to the α1β2 dimer. Subsequent binding of molecular oxygen to the four heme groups drives the Hb tetramer from the deoxy **T** to the oxy **R** state, rendering the **T**→**R** transitions the molecular process that underlies the well-known cooperativity of ligand binding. Despite the wealth of structural information available for Hb, the mechanistic coupling between the quaternary transition and the tertiary transitions in the individual subunits remains poorly understood. We report spontaneous and reproducible **T**-**R** transitions of Hb in molecular dynamics simulations, allowing us to study the mechanism underlying the transitions in atomistic detail. We pay special attention to the interplay between the tertiary and quaternary transitions of Hb, as well as to the tertiary **t**/**r** populations of the subunits in a particular (**T** or **R**) quaternary state. Interestingly, we observe a pronounced asymmetry between the α and β subunits with the β subunits being more strongly linked to the quaternary transitions than the α subunits. The simulations allow us to propose a pathway for coupled quaternary and tertiary transitions between the **R** and **T** conformations of Hb.

## Introduction

Conformational transitions of allosteric proteins are fundamental to a variety of biological functions. For instance, quaternary transitions in hemoglobin (Hb) give rise to the cooperativity of ligand binding and have therefore drawn extensive and ongoing scientific interest over many decades [1], [2]. The end points of the quaternary transition of Hb are referred to as deoxy **T** state and oxy **R** state of Hb, which are characterized by low and high oxygen affinity, respectively [3], and the cooperativity of ligand binding originates from the dependence of quaternary population on the number of liganded subunits [4]. The oxygen affinity of Hb decreases with lower pH, a phenomenon that is referred to as alkaline Bohr effect. Approximately 40% of the Bohr effect has been attributed to the protonation of the terminal His146 residues of the β subunits, which are also denoted as ‘HC3 histidines’ [5].

**Figure 1:**
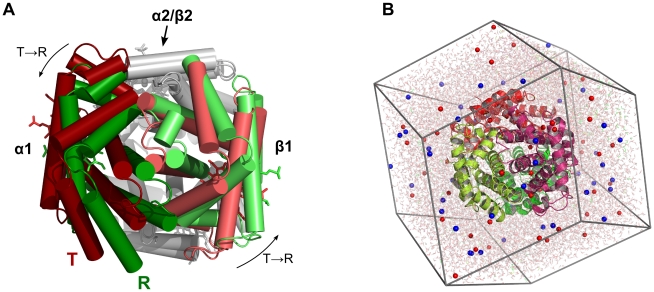
Human Hemoglobin (Hb). (A) X-ray structures of Hb in the **R** state (green) and in the **T** state (red). The **T**-**R** quaternary transition is mainly characterized by a rotation of the α1/β1 dimer (colored) with respect to the α2/β2 dimer (gray). To visualize the rotation, the α2/β2 dimers of the **R** and **T** structure were superimposed on each other. (B) A typical dodecahedral simulation box of Hb. The Hb tetramer is shown in cartoon representation, sodium and chloride ions as red and blue spheres, respectively, and water is depicted as transparent sticks. The molecular representations were made with Pymol [48].

The stereochemical explanation of Hb cooperativity and the characterization of the transition pathway were originally based on the HbCO and deoxyHb crystal structures, corresponding to the **R** and **T** state, respectively. According to these structures, the transition can mainly be described by a 12–15° rotation of the α1β1 dimer with respect to the α1β2 dimer (Fig. 1A) [1], [6]. Later, a second quaternary structure of liganded Hb, termed **R2**, was found [7], with a 1.1Å larger distance between the centers of mass (COMs) of two β subunits as compared to the **R** structure. Differences between **R** and **R2** at the α1–β2 interface triggered a still unresolved discussion whether **R2** is a stable intermediate on a **R**-**R2**-**T** pathway [7]–[9]. NMR experiments indicate that liganded Hb in solution is in equilibrium between the **R** and **R2** structures [10]. More recently, two additional liganded Hb structures **RR2** and **R3** were found using the high-salt crystallization conditions of Perutz [11], emphasizing that a consensus view on the liganded Hb state in solution is far from being reached. **RR2** represents an intermediate structure between **R** and **R2**, whereas the distance between the COMs of the two β subunits is reduced by 3.1Å in **R3** as compared to the **R** structure.

Extensive efforts aimed to identify the transition pathway of Hb in response to ligand dissociation [12]. The kinetics of the **R**→**T** transition after photodissociation of the CO adduct, HbCO, have been studied using time-resolved spectroscopic techniques including absorption [13], Raman [14], [15], and circular dichroism spectroscopy [16], [17]. The picture derived from these experiments suggests a multistep **R**→**T** pathway via several metastable intermediates, with relaxation rates ranging from tens of nanoseconds to tens of microseconds, and with a time constant of ∼21µs for the overall **R**→**T** quaternary transition [15]. The experiments provide extremely valuable insights into the kinetics of Hb, but they also bear limitations. They do not directly detect the global quaternary transitions, but mainly measure the formation of hydrogen bonds of aromatic residues, such as the Trp(β)37-Asp(α)94 and the Tyr(α)42-Asp(β)99 H-bonds, which must be interpreted in terms of conformational transitions. A full-atomistic picture of the **R**→**T** transition could so far exclusively be derived for a mollusk dimeric hemoglobin using time-resolved X-ray crystallography [18]. Such experiments provide an ensemble-averaged picture, whereas Hb may follow heterogeneous transition pathways that may not be fully reflected by the spectra. Furthermore, in contrast to the well-studied **R**→**T** transition, little is known about the kinetics of the **T**→**R** transition because that transition cannot be triggered by photolysis.

Molecular dynamics (MD) simulations can provide a full-atomistic picture of Hb and are therefore well suited to complement experimental efforts. Early MD efforts focused on the photodissociation of CO [19], or were restricted to the dynamic treatment of a subset of Hb residues [20]. Ramadas and Rifkind considered the response of Hb to the perturbation of the heme on a several 100ps time scale [21], and Mouawad and coworkers enforced quaternary transitions within 200ps using a technique called path exploration method [22]. In addition, a set of MD simulation of up to 6ns were carried out with a focus on the mechanism of effectors [23]. Recently, a single 45-ns simulation of Hb was published without observing any conformational transitions [24]. Complementary to the MD studies, a normal mode analysis considered the collective motions intrinsic to the Hb tetramer [25], and an elastic network study suggested a **T**-**R2** transition as the preferential quaternary transition pathway [9].

So far, no spontaneous quaternary or tertiary transitions of Hb were observed during MD simulations, presumably since previous simulations were restricted to too short time scales. Here, we apply extensive MD simulations to investigate the deoxy **R**, **R2**, and **T** state of human. We observe for the first time spontaneous and reproducible quaternary transitions of Hb, as well as tertiary transitions of the α and β subunits. Hence, these simulations allow one to study the transition mechanism in atomistic detail. We find the **T**-**R** (in contrast to the **T**-**R2**) pathway as the primary quaternary transition pathway. By analyzing repeated **T**-**R** transitions, we find a marked asymmetry between the α and β subunits. Based on the simulation results, we present a schematic mechanism underlying the preferential transition pathway between the **R** and **T** states of hemoglobin.

## Results

For the present study, Hb was simulated using five different initial configurations. (1) Starting from the **R**-state with His(β)146 protonated (HC3 protonation), (2), from the **R**-state with His(β)146 deprotonated, (3) from the **T**-state with His(β)146 protonated (HC3 protonation), and (4) from the **T**-state with His(β)146 deprotonated, and (5) starting from the **R2** state with deprotonated His(β)146. All simulations are summarized in Table 1. In a first step, we aimed to assess whether Hb displays spontaneous quaternary transitions within the simulated times scales of several hundred nanoseconds. To this end, each of the configurations 1–3 was simulated three times for 200ns starting with different initial velocities, and configuration 4 was simulated six times for 300ns. Simulations starting from **R2** were carried out three times for 130ns. Together with 20 additional 50ns simulations starting from **T** and simulations with restrained salt bridges (see below), the simulation times sum up to a total of ∼5.6 µs of Hb simulation, which is at least 2 orders of magnitude longer than simulations in previously reported MD studies of Hb [21]–[24].

**Table 1:** Summary of molecular dynamics simulations of human hemoglobin A carried out for the present study.

Initial structure	His(β)146 protonated	Time [ns]	# carried out	Salt bridges restrained	Name	Figures
**R**	Yes	200	3x	-	R.HC3-x	2
**R**	-	200	3x	-	R.noHC3-x	S1
**R2**	-	130	3x	-	R2-x	S2
**T**	Yes	200	3x	-	T.HC3-x	3, 4
**T**	-	300	6x	-	T.noHC3-x	S3
**T**	-	50	20x	-	-	5, S4
**T**	Yes	200	3x	During the first 100ns	T.SBres-x	S5

### Simulations starting from R or R2

The simulations starting from the **R** or **R2** were carried out without carbon monoxide (CO). Removal of CO from the structures is expected to shift the equilibrium between the **T** and **R** states towards the deoxy **T** state, corresponding to a CO photodissociation experiment. To quantify the state of Hb with respect to the **R**, **R2**, and **T** state, a principal component analysis (PCA) was applied to the X-ray structures of the **R**, **R2**, and **T** states [7], [26], [27]. The PCA was carried out using the C-α and the heme group atoms. The resulting two X-ray eigenvectors span the **R**-**R2**-**T** subspace and hence, projections onto these two eigenvectors can visualize transitions between the **R**, **R2**, and **T** states. The linear motions along the two eigenvectors are also visualized in video S1. The first eigenvector is mainly characterized by a large-scale rotation of the α1β1 relative to the α2β2 dimer as required for the **T**-**R** quaternary transition, whereas the second eigenvector accounts for the distance between the two β subunits.

**Figure 2:**
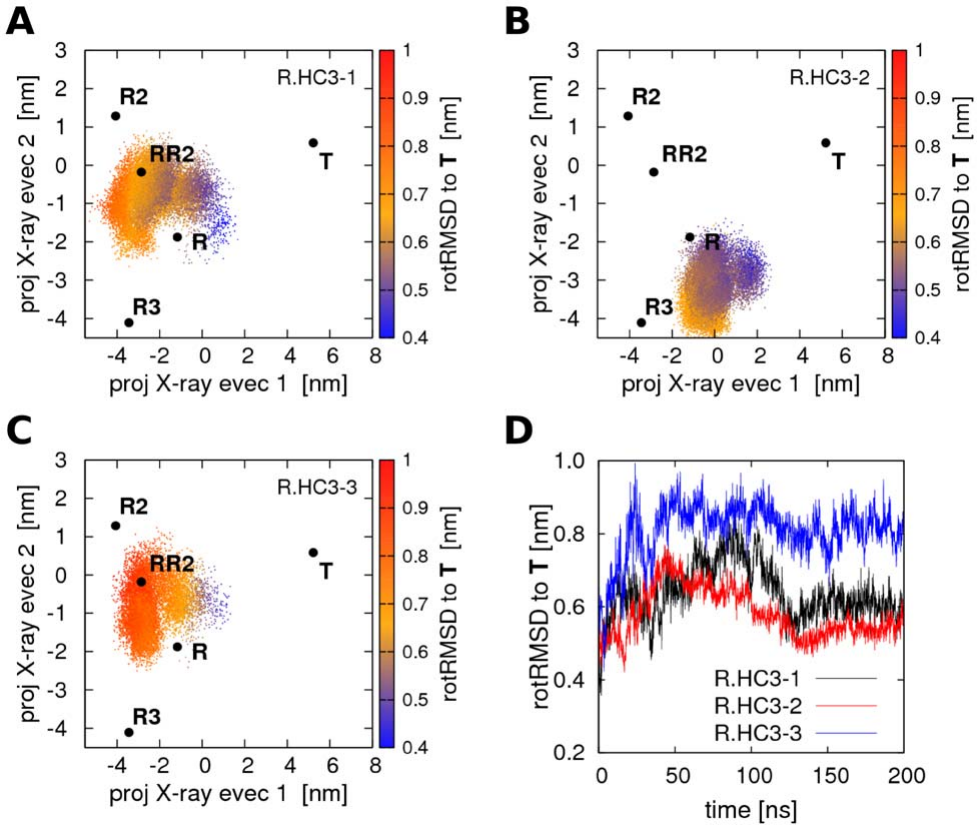
PCA projections of Hb simulations starting from R with protonated His(β)146. (A–C) Projections of Hb structures during simulations R.HC3-1 to R.HC3-3 on the two eigenvectors derived from a PCA of the **T**, **R**, and **R2** X-ray structures. The **T**, **R**, **R2**, **RR2**, and **R3** X-ray structures are indicated by black dots. The color encodes the rotRMSD with respect to the **T** X-ray structure. (D) rotRMSD of simulations R.HC3-1 to R.HC3-3 with respect to the **T** X-ray structure. No **R**→**T** transition occurs within simulation time, but simulations R.HC3-1 and R.HC3-3 approach the **RR2** structure on a sub-100-nanosecond timescale.

In all simulations starting from the **R** state with HC3 protonation (denoted R.HC3-1 to R.HC3-3), starting from the **R** state without HC3 protonation (denoted R.noHC3-1 to R.noHC3-3) or from the **R2** state (denoted R2-1-R2-3), no quaternary transitions to the **T** state occurred during 200ns or 130ns of simulation, respectively. The PCA plots of the simulations R.HC3-1 to R.HC3-3 are shown in Fig. 2A–C. The **R**, **R2**, and **T** as well as the more recently resolved **RR2** and **R3** structures [11] are indicated by black dots, and the simulation structures by colored dots. All three simulations R.HC3-1 to R.HC3-3 remain in the proximity of the **R** state, but clearly deviate from the **R** X-ray structure. Noteworthy, R.HC3-1 and R.HC3-3 sample the region between the **R** and **R2** structure, that is the conformational space near the **RR2** X-ray structure (PDB code 1MKO), showing that transitions between conformations around the **R** and **RR2** structures can occur on a 100ns time scale. In contrast none of the simulations starting from **R** approach the **R3** structure (PDB code 1YZI).

The inability of the simulations to reach the **T** state in the simulated time is also visible from the root mean square deviation (RMSD) plot in Fig. 2D. The RMSD has been computed by first superimposing the α1β1 dimer onto the α1β1 dimer of the **T** X-ray structure. Subsequently, the backbone RMSD of the α2β2 dimer with respect to the α2β2 dimer of the **R** X-ray structure was computed, providing a measure which is particularly sensitive to the rotation of the two dimers with respect to each other and hence, to the **R**→**T** quaternary transition. This RMSD measure has been applied previously [7], and it is henceforth referred to as ‘rotRMSD’. Note that the rotRMSD yields larger values than the standard RMSD measure that is typically computed after superimposing the complete structure onto the reference. For instance, the backbone rotRMSD between the **R** and **T** X-ray structures equals 0.53nm, whereas the standard backbone RMSD is 0.24nm. As visible from Fig. 2D, none of the three simulations approaches the **T** state, with the rotRMSD being larger than the rotRMSD between the **R** and **T** X-ray structures. The corresponding plots for the three simulations starting from **R** without HC3 protonation (R.noHC3-1 to R.noHC3-3) as well as for the three simulations starting from **R2** (denoted R2-1 to R2-3) are shown in supporting Fig. S1 and S2, respectively. No spontaneous quaternary transitions to the **T** state occurred during these simulations. Simulation R2-3 temporarily samples conformations near the **RR2** structure (Fig. S2C), but no full transitions between **R** and **R2** were observed (Fig. S1 and S2). Taken together, the simulations starting from **R** or **R2** remain in the conformational space around the **R**, **RR2**, and **R2** structures within hundreds of nanoseconds. On the same time scale, simulations starting from **R** or **R2** occasionally sample conformations around the **RR2** structure as measured from the eigenvector projections, suggesting that **R**, **RR2**, and **R2** can be interpreted as representatives of a larger R-like ensemble.

### T→R transitions

**Figure 3:**
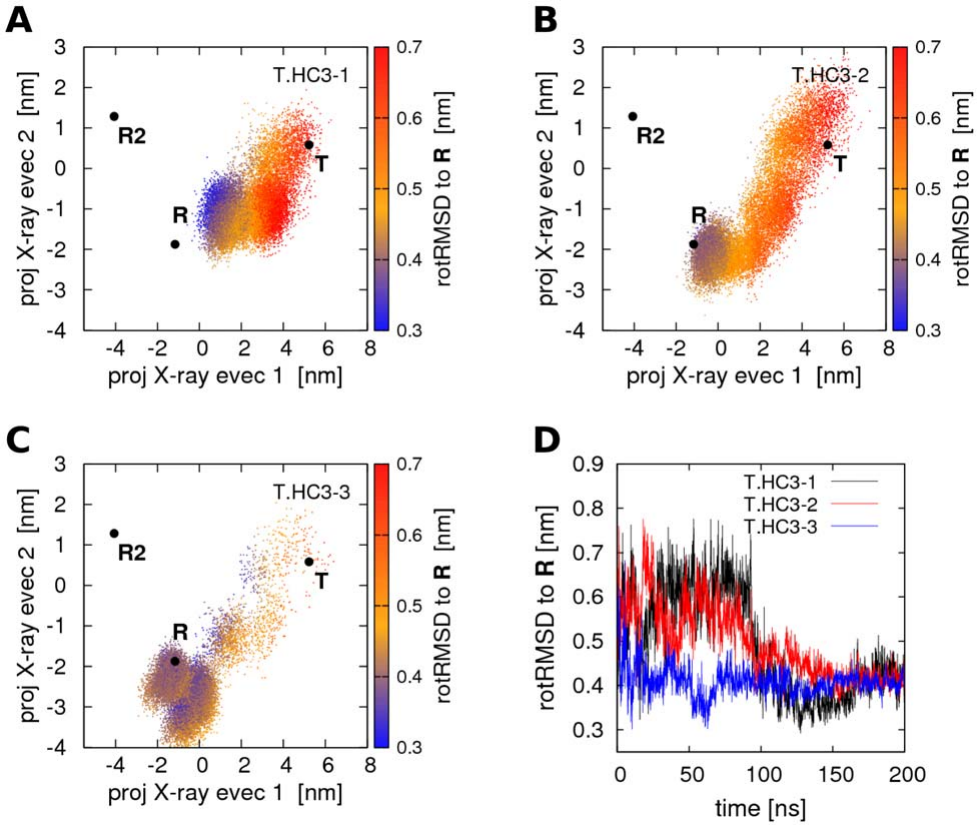
Quaternary T→R transitions in Hb simulations with protonated His(β)146. (A–C) Projections of Hb structures during simulations T.HC3-1 to T.HC3-3 on the two eigenvectors derived from a PCA of the **T**, **R**, and **R2** X-ray structures. The color encodes the rotRMSD to the **R** X-ray structure. Full quaternary transitions of Hb occur during simulation time. (D) rotRMSD of simulations T.HC3-1 to T.HC3-3 to the **R** X-ray structure.

In contrast to the rather stable simulations starting from **R** or **R2**, the simulations starting from the **T** state show a remarkable and reproducible tendency towards the **R** state. In the following, the three simulations starting from **T** with protonated His(β)146 are denoted as T.HC3-1 to T.HC3-3. Figures 3A–C show the projections of T.HC3-1 to T.HC3-3 onto the two X-ray eigenvectors. The **R**, **R2**, and **T** are indicated by black dots and the simulation structures as colored point clouds. The color indicates the rotRMSD of each simulation structure with respect to the **R** state and has again been measured by first superimposing the α1β1 dimer and subsequently computing the RMSD from the α2β2 dimer only. The rotRMSD during the simulations T.HC3-1 to T.HC3-3 is also plotted in Fig. 3D. During all three simulations, Hb closely approaches the **R** state, as either measured from the PCA vector projections or from the rotRMSD. Note that the rotRMSD to **R** stabilizes at values ≤0.4nm, close to the rotRMSD of the simulations which started from **R** and which remained in the proximity of **R** (Fig. S1A–C and S1E). Hence, simulations T.HC3-1 to T.HC3-3 describe complete spontaneous quaternary transitions from **T** to **R**. The simulation time required for the quaternary transition substantially differs between the three simulations. Whereas T.HC3-1 and T.HC3-2 require 100–150ns, T.HC3-3 approaches **R** up to a rotRMSD of 0.35nm within only 2ns, although additional 20ns are required before Hb is stabilized in the proximity of **R**. To our knowledge, T.HC3-1 to T.HC3-3 are the first reported simulations of full spontaneous quaternary transitions of Hb. Noteworthy, during none of the simulations starting from **T**, Hb approached the **R2** structure, rendering any role of **R2** as an intermediate between **T** and **R** unlikely, in agreement to previous geometrical considerations [8]. The transition in simulation T.HC3-3 is also visualized in video S2.

The HC3 histidines have been shown to contribute ∼40% to the alkaline Bohr-effect [5] and hence, the deprotonation of HC3 is expected to (further) destabilize the **T** state and drive Hb towards the **R** state. To assess whether this effect can be observed in the simulations, we have carried out six 300-ns simulations starting from the **T** state, this time with the His(β)146 being deprotonated (referred to as T.noHC3-1 to T.noHC3-6). The projections of the structures from T.noHC3-1 to T.noHC3-6 onto the X-ray eigenvectors are plotted in Fig. S3A–C/E-G. Likewise to simulations with double-protonated His(β)146, some of the T.noHC3 simulations carried out full or partial quaternary transitions from **T** to **R**, as visible from the eigenvector projections (Fig. S3A/F/G) as well as from the rotRMSD to **R** (e.g., black curve in Fig. S3D). In contrast, T.noHC3-2 and T.noHC3-3 were not able to carry out the **T**→**R** transition within simulation time, although they also showed a tendency towards **R** at the beginning of the simulations. Visual inspection of the trajectories show that the lack of a positive charge at the His(β)146 indeed accounts for this result. If His(β)146 are deprotonated, the lack of salt bridges renders His(β)146 more flexible as compared to the protonated His(β)146 in the T.HC3 simulations. As a result, the His(β)146 frequently entered the junction between the two β subunits (as present in the **R** X-ray structure), but one (or both) of the two His(β)146 alternatively formed hydrogen bonds to the nearby α subunit, blocking the transition towards the **R**-state for the remaining several hundred nanoseconds of the simulations. In contrast, the protonated histidines (simulations T.HC3-1 to T.HC3-3) were found to be structurally stable near the His(β)146 position of the **T** X-ray structure, tightly bound to Asp94 and Glu90 by subunit-internal salt bridges. Therefore, these histidines do not interact with the nearby α subunit, allowing the two β subunits to rapidly approach each other as required for the **T**→**R** transition. Hence, deprotonation of His(β)146 results in opposing consequences. On the one hand, it allows the two His(β)146 to enter the junction with the imidazole rings pointing towards each other, as present in the **R** X-ray structure. On the other hand, uncharged His(β)146 are flexible and thus form metastable configurations which inhibit the **T**→**R** quaternary transition for at least hundreds of nanoseconds.

### Rigid-body rotations during T→R transitions

**Figure 4:**
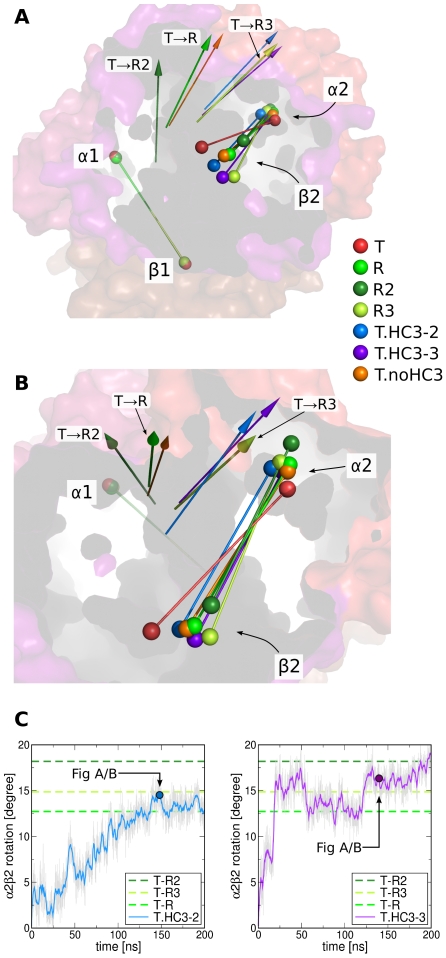
Rotation of the α2/β2 dimer with respect to the α1/β1 dimer during T→R transitions. (A/B) Colored rods indicate the dimers and the spheres represent the center of mass (COM) of the four subunits as labeled in the panels A/B. Before analyzing the rotation of the α2/β2 dimer, the α1/β1 dimer of the structures were superimposed on the α1/β1 dimer of the **T** X-ray structure (red rods and transparent surface). The **R**, **R2**, and **R3** X-ray structures are colored in light green, dark green, and lime green, respectively (compare legend). Representative structures of simulations that displayed **T**→**R** transitions are presented as blue, violet, and orange rods. The colored arrows indicate the rotation axes that map the **T** α2/β2 dimer (red rod) onto the dimer of the respective X-ray or simulation structure. The colors of the arrows correspond to the colors of the dimer rods. (C) Rotation of the axis connecting the center of mass of the α2 and β2 subunits during simulations T.HC3-2 and T.HC3-3. The angle is computed with respect to the respective axis in the **T** X-ray structure. The angles between the **T** and the **R**, **R2**, and **R3** X-ray structures are indicated by dotted lines. The simulation snapshots corresponding to the blue and violet rods in panels A/B are indicated by colored dots.

Complementary to the PCA projections and the rotRMSD measure, we have analyzed the quaternary **T**→**R** transition in terms of rigid-body rotations of the α2β2 dimer with respect to the α1β1 dimer. Accordingly, we quantified the rotation of the dimeric α2β2 axis that connects the center of mass (COM) of the α2 subunit with the COM of the β2 subunit (colored rods in Fig. 4A/B). In line with the rotRMSD calculation, the rotation of the α2β2 axis was measured by first superimposing the α1β1 dimer onto the α1β1 dimer of the **T** X-ray structure. Subsequently, the angle between the α2β2 axis and the α2β2 axis of the **T** X-ray structure was calculated.

Figures 4A/B present the dimeric axes of the **T** X-ray structure as red rods, where the red spheres indicate the COMs of the subunits. The blue, violet, and orange rods show the α2β2 axis of snapshots from simulations that (partially) carried out the **T**→**R** transition. Snapshots represented by the blue and violet rods were taken from simulations T.HC3-2 and T.HC3-2 (compare Fig. 3B/C), and the orange rod from one of the 20 additional 50ns-simulations starting from **T** (with deprotonated HC3 histidines) that closely approached the **R** structure (corresponding to the black curve in Fig. S4). For comparison, the α2β2 axes of the **R**, **R2**, and **R3** structures are shown as light green, dark green, and lime green rods, respectively. In line with the PCA projections (Fig. 3), the α2β2 dimer displays a rotation from the **T** orientation to the **R** orientation during theses simulations. In addition, the COMs of the α2 and β2 subunits (spheres in Fig. 4A/B) approach the respective COMs in the **R** structure or, alternatively, take an intermediate position between COMs in the **R** and **R3** structures (violet rod). In none of these simulations, the α2β2 axis approaches the respective position in the **R2** structure (dark green). Figure 4C plots the α2β2 rotation angle versus time during simulations T.HC3-2/3, confirming the rotational **T**→**R** transition.

The arrows in Fig. 4A/B indicate the rotation axes that map the **T** α2β2 axis onto the α2β2 axes in the simulation snapshots or onto the axes in the **R**, **R2**, and **R3** structures. According to these arrows, the rotation axis during the T.noHC3 simulation (orange arrow) resembles the **T**→**R** rotation axis, whereas the rotation axes in simulations T.HC3-2/3 resemble the **T**→**R3** rotation axis. Hence, the **T**→**R** transition is not restricted to a single rotation axis, suggesting that the quaternary transition can instead occur via different rotational (and translational) degrees of freedom.

### Interplay between quaternary and tertiary conformational transitions

The simulations displaying full **T**→**R** transitions allowed us to probe the interplay between quaternary and tertiary transitions. A reaction coordinate for the quaternary transition is given by the projection onto the difference vector connecting the **T** and **R** X-ray structures. Similarly, projections onto the difference vectors representing the tertiary **t**-**r** transitions of the α and β subunits were used as reaction coordinates for the tertiary conformational transitions. Here, the lower-case letters **r** and **t** denote the subunit conformations in the **R** and **T** X-ray structures, respectively. As shown in Fig. S7, the projection onto the quaternary transition vector is strongly correlated to the dimer rotational angle during **T**→**R** transitions, suggesting that the projection on the quaternary transition vector can be interpreted as the dimeric rigid-body rotation analyzed in the previous paragraph.

**Figure 5:**
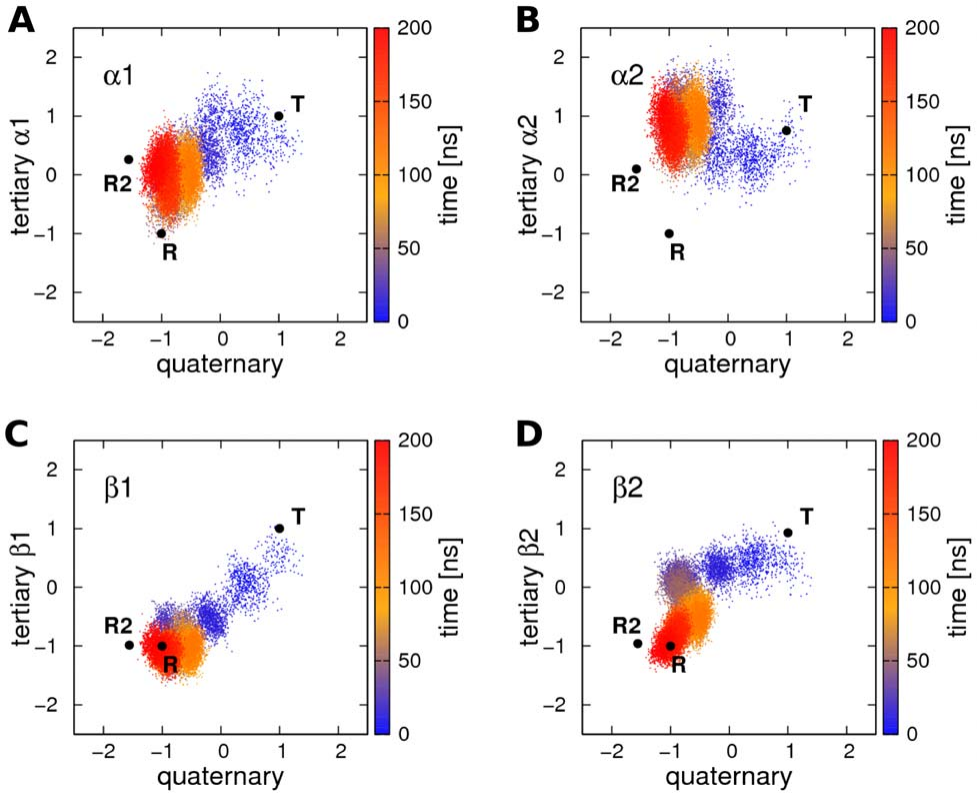
Tertiary versus quaternary transition during simulation T.HC3-3 (compare **Figure 3C**). Projection onto the quaternary transition vector connecting **R** and **T** X-ray structures (x-axis) is plotted versus the projection onto the tertiary transition vectors connecting the **r** and **t** structures of the (A) α1, (B) α2, (C), β1, and (D) β2 subunit. The projections were normalized such that −1 corresponds to the **r**/**R** state, and +1 to the **t**/**T** state. The color indicates the simulation time. The quaternary transition occurs simultaneously to the tertiary transition of the β subunits.

Figure 5 displays the projections of simulation T.HC3-3 on the tertiary versus the quaternary **T**-**R** transition vectors. The projections are normalized such that the **T**/**t** state (quaternary/tertiary) corresponds to a projection of +1, whereas the **R**/**r** state corresponds to a projection of −1. Before the normalization applied for Fig. 5, tertiary projections of ±1 correspond to projections of ±0.76nm and ±0.92nm for the α and β subunits, respectively. Thus, the β subunits undergo a larger internal rearrangement during **t**-**r** transitions as compared to the α subunits. The color-coding represents simulation time. Interestingly, the tertiary transitions of the β subunits are correlated to the quaternary transition in that simulation. The tertiary transition of β1 occurs simultaneously to the quaternary transition during the first 20ns (blue dots in Fig. 5C), and the final step in the quaternary transition between ∼100ns and ∼200ns occurs simultaneously to the final step in the transition of β2 (yellow and red clouds in Fig. 5D). In contrast, no such correlation can be observed between the quaternary transition and the transition of the α subunits.

**Figure 6:**
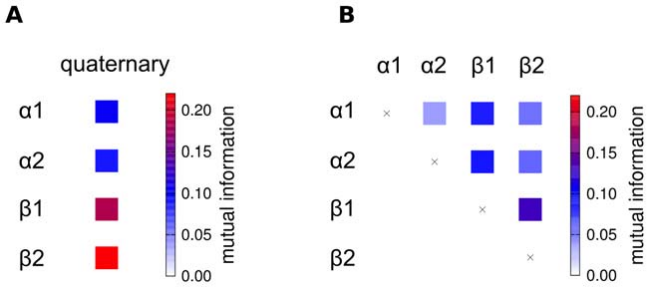
(A) Correlation between quaternary and tertiary transitions as averaged from six independent **T**→**R** transitions, and (B) correlation between subunits during **T**→**R** transitions. The correlation is measured using the mutual information (MI) between the projections on the respective difference vectors connecting the **T** and the **R** X-ray structures.

To assess if the observed correlation between subunits and the Hb quaternary structure is a robust feature of **T**→**R** transitions, we have carried out 20 additional 50-ns simulations of Hb starting from the **T** state with deprotonated His(β)146. In six of the 20 simulations, Hb carried out a full transition to the **R** state, as detected from a rotRMSD of <0.3nm to the **R** X-ray structure (Fig. S4). We applied the so-called mutual information (MI) to measure the correlation between the quaternary and tertiary transitions during these six **T**→**R** transitions. MI is a quantity from information theory measuring correlation between two arbitrary variables. MI does not only detect linear correlation, but quantifies any kind of interdependence including non-linear and higher-order correlation. Figure 6A depicts the MI between quaternary and tertiary transitions, as averaged from the six transitions observed in the 50-ns simulations. In line with simulation T.HC3-3 (Fig. 5), the quaternary transition is substantially stronger correlated to the tertiary transitions of the β subunits than to the tertiary transitions of the α subunits.

The average MI between the subunits during the **T**→**R** transitions is visualized in Fig. 6B. Clearly, the correlation between any pair of the subunits is significantly weaker than the correlation between the β subunits and the quaternary transition (compare Fig. 6A). Only the two β subunits are to some extent correlated to each other. That correlation may, however, arise indirectly via the correlation of each of the β subunits to the quaternary transition. Taken together, the tertiary transition of the β subunits occur simultaneously to the quaternary **T**→**R** transition, whereas the transitions of the α subunits can occur on longer time scales after completion of the quaternary transition.

### Tertiary populations in a given quaternary state

Before the identification of functionally different tertiary states in the **T** quaternary structure [28], [29], allosteric models for Hb did not explicitly consider the population of different tertiary states of Hb subunits within a given quaternary state. Instead, the cooperative effects in Hb were attributed to the dependence of subunit oxygen affinity on the quaternary state of Hb, whereas at the same time, the population of the quaternary states depends on the number of liganded subunits (Eaton et al, 2007). These models include the quaternary two-state model by Monod, Wyman, and Changeux (MWC [4]), the Cooperon model by Brunori and coworkers [30], [31], as well as the model by Szabo and Karplus, which was later generalized by Lee and Karplus [32], [33]. More recently, Henry and coworkers proposed a tertiary two-state (TTS) model to account for alterations in the ligand affinity in the **T** quaternary state by allosteric effectors [34]. According to the TTS model, the subunits in the **T** quaternary state can populate both the **r** *and* **t** tertiary states. Likewise for Hb in the **R** quaternary state, the subunits can adopt both the **r** and **t** tertiary states.

**Figure 7:**
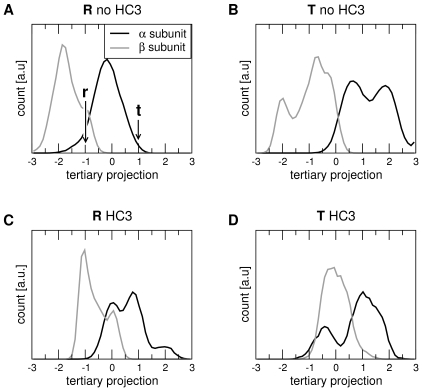
Populations of tertiary states of the α (black) and β (grey) subunits as a function of quaternary state (T/R) and the HC3 protonation. The tertiary states were computed from the projections of the subunit structures during simulation onto the tertiary difference vectors connecting the **T** and **R** tertiary X-ray structures, denoted by **t** and **r**. The projections were normalized such that the **r** and **t** tertiary structure correspond to a projection of −1 and +1, respectively. A simulation frame was assigned to the **R** or to the **T** quaternary structure, if the projection onto the vector connecting the **R** and **T** quaternary states was <−0.5 or >+0.5, respectively (compare Fig. 5). Populations in Hb simulations without deprotonated His(β)146 are denoted by ‘**T**/**R** no HC3’ (A/B), and simulations with protonated His(β)146 are denoted by ‘**T**/**R** HC3’ (C/D).

The populations of the tertiary states **r** and **t** within each of the quaternary states **R** and **T**, as derived from the MD simulations, are presented in Fig. 7. The figure plots the probability distributions of the projections of the α (black) and β (grey) subunits onto the difference vector connecting the **r** and **t** X-ray structures. In line with Fig. 5, the projections in Fig. 7 are normalized such that −1 and +1 correspond to **r** and **t**, respectively. A simulation frame has been assigned to the **R** or **T** quaternary state, if the projection onto the difference vector connecting the **R** and **T** X-ray structures was smaller −0.5 or larger +0.5, respectively (where projections of −1 and +1 again correspond to the **R** and **T** quaternary state, respectively). The analysis has been carried out for simulations of Hb with deprotonated His(β)146 (Fig. 7A/B) and for simulations with protonated His(β)146 (Fig. 7C/D).

Although the populations are presumably not fully converged within the simulation timescales, a number of significant conclusions can be drawn from Fig. 6. (i) Within a given quaternary state (**R** or **T**), the α and β subunits populate a continuous ensemble of substates, including both the **r** and **t** tertiary states known from the X-ray structures, in line with the idea of the TTS model that different tertiary states populate a given quaternary state. However, the α and β subunit ensembles are not constricted to the region between the respective tertiary X-ray structures (at projections of −1 and +1), but the subunits in addition show a high probability to populate states “beyond” the X-ray structures, at values below −1 and above +1 (ii) The distributions of the α and β subunits are not equivalent. The β subunits (grey curves) display a strong tendency towards the **r** structure, even for the Hb tetramer in the **T** quaternary state (Fig. 7B/D). In contrast, the α subunits (black curves) tend towards the **t** structure, even for the tetramer in the **R** quaternary state (Fig. 7A/C). (iii) As expected, generally the tertiary subunit distributions in the **R** quaternary state are shifted towards the **r** tertiary state, and the subunit distributions in the **T** quaternary state are shifted towards the **t** tertiary state (compare Fig. 7A to B and C to D). (iv) A comparison of Fig. 7 A/C to C/D shows that the protonation of His(β)146 shifts the populations of the β subunits from the **r** towards the **t** state (to the right in Fig. 7), whereas the α subunits are relatively unaffected by the protonation. This finding is to some extend expected because the His(β)146 are located at the β1/β2 interface, mainly affecting the β subunits. Note that the shift in the β population towards **t** is also in accordance with the alkaline Bohr effect, that is with the tendency towards the low-affinity **T** state in a more acidic environment.

## Discussion

We have reported reproducible spontaneous quaternary transitions of human Hb A from the **T** to the **R** state in atomistic molecular dynamics simulations, as well as tertiary transitions of the Hb subunits. The simulations draw a highly diverse picture of the **T**→**R** transitions. The **T**→**R** transition occurs on different time scales, depending on the pathway of the transitions and on the stability of metastable conformations formed during the transitions. For example, the conformation characterized by hydrogen bonds between His(β)146 and the nearby α subunit was frequently formed in simulations starting from the **T** state with deprotonated His(β)146, slowing down the transition by at least hundreds of nanoseconds. Noteworthy, the simulations starting from the **T** X-ray structure frequently approached the **R** but never the **R2** X-ray structure, in contrast to a previous elastic network study that reported a tendency of **T** towards the **R2** state [9]. Simulations starting from **R** or **R2** did not approach **T** during simulation time, as expected from spectroscopic measurements that suggest a 21µs relaxation time for the full **R**→**T** transition [15]. Instead, transitions between **R** and **RR2** or **R2** and **RR2** occurred on a 100ns timescale, suggesting that these structures can be interpreted as three representatives of a continuous R-like ensemble, in accordance with previous NMR studies that suggested a dynamic equilibrium between the **R** and **R2** X-ray structures [10]. Our simulations would also be line with a **T**-**R**-**R2** allosteric pathway as suggested from geometrical considerations and crystallographic studies [8], [35]. Noteworthy, the trajectories of Hb starting from the same structure (**T**, **R**, or **R2**) typically diverge, sampling different parts of the phase space even on the hundreds of nanoseconds time scale. This finding emphasizes (i) the diversity of conformations accessible to Hb, and (ii), that single MD trajectories must be interpreted with care because they may not represent the complex behavior of Hb.

A surprising result of our simulations is the instability of the **T** state and its remarkable tendency towards **R**. Because we have simulated unliganded Hb, we had expected Hb to be stable in its deoxy **T** state, in particular with protonated His(β)146. A possible artifact from the applied force field seems unlikely, because data from previous computational studies using different force fields also suggest a relatively instable **T** state. Mouawad and Perahia observed a low-barrier quaternary pathway from **T** towards a quaternary **R**-like state derived from a combination of normal mode analysis and energy minimization using the QUANTA, CHARMm21, and CHARMM22 force fields. [25]. A 6-ns simulation of Hb starting from **T** (using the CHARMM force field) reported by Laberge and Yonetani displayed a rapidly increasing backbone RMSD up to 4Å [23]. In addition, Saito and Okazaki published a single 45-ns trajectory starting from an oxy T-state (pdb 1GZX [36]) using the AMBER96 force field. In that simulation, the distance between the α1β1 dimer and the α2β2 dimer increased by 2 Å within only 15ns. That displacement may, however, also originate from the oxygenated heme groups present in the 1GZX structure.

To further investigate the instability of the **T** state, we have carried out three additional 200-ns simulations of Hb starting from **T** with protonated His(β)146 (termed T.SBres-1 to T.SBres-3, Fig. S5). During the first 100ns of these simulations, all salt bridges which are present in **T** but broken in the **R** state were restrained by applying a harmonic potential between the respective salt bridge partners (see experimental procedures for details). After 100ns, the salt bridge restraints were removed and each of the three systems was simulated for additional 100ns. Two putative sources of artifacts were addressed with these simulations: (i) could present-day force fields underestimate the stability of the salt bridges, leading to an unstable **T** state? And (ii) could non-equilibrium effects, such as a non-equilibrated electrolyte distribution, destabilize **T**? Both sources of artifacts could be excluded from simulations T.SBres-1-3. (i) Although full **T**→**R** transitions cannot occur during the first 100ns of these simulations, they showed the same reproducible tendency of **T** towards **R** (Fig. S5A–C, green dots), suggesting that a putative lack of salt bridge stability does not induce the tendency of **T** towards **R**. (ii) After 100ns of simulation with restrained salt bridges, the systems represent well-equilibrated T-like structures. However, after releasing the salt bridges, these simulations carried out transitions towards **R** as well (Fig. S5A–C, magenta dots), and T.SBres-3 even approaches the **R** X-ray structure as measured from a rotRMSD <0.4nm (Fig. S5D). Hence, limited equilibration can be excluded as a reason for **T**→**R** transitions. Taken together, our simulations together with previous computational studies suggest that its instability and tendency towards **R** is an inherent property of the Hb **T** X-ray structure. However, we also note that further studies will be required to fully rationalize that finding.

**Figure 8:**
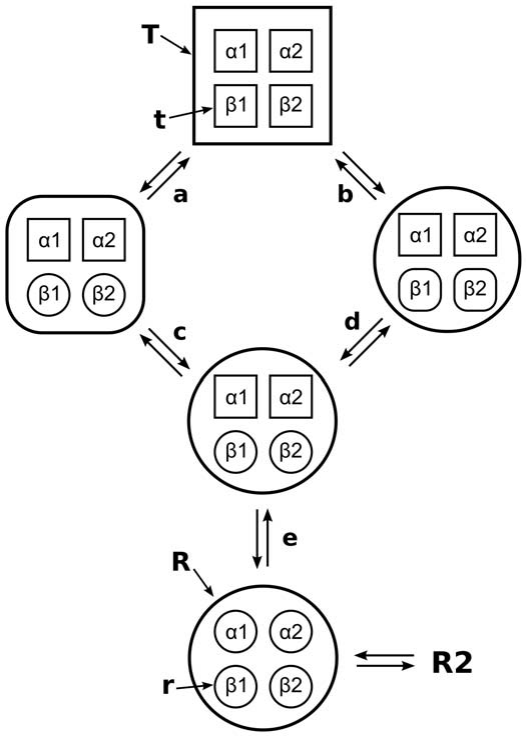
Schematic representation of consensus transition pathways between the R and T state as derived from the MD simulations. The large sphere and square depict the quaternary **R** and **T** states, respectively, whereas the small spheres and squares depict tertiary **r** and **t** states, respectively. A square with round corners indicates an intermediate structure between **R** and **T** or between **r** and **t**.

We have analyzed the correlation between the quaternary and tertiary conformational transitions during six independent **T**→**R** transitions, as measured from the mutual information. The analysis shows that, during the **T**→**R** transition, the β subunits are substantially stronger coupled to the quaternary transition than the α subunits. The internal transitions of the four subunits seem relatively uncorrelated, with the exception of a slight correlation between the β subunits, which may however originate from the coupling from each of the β subunits to the quaternary transition, rather than from a direct coupling between the β1 and β2 subunits. Together with the tertiary distributions in a given quaternary state (Fig. 7), these results allow one to extract a consensus **T**-**R** transition pathway from the simulations, as shown in Fig. 8. Squares in Fig. 8 stand for **T**/**t** structures, circles for **R**/**r** structures, and squares with round corners for intermediate structures between **T**/**t** and **R**/**r**. We stress that these symbols represent structural similarity to the X-ray structures (or between them), and may not be equivalent to the low-affinity and high-affinity states present in solution. In simulations starting from the **T** X-ray structure (top in Fig. 8), two possible pathways towards the quaternary **R** structure were observed. (a) A *partial* quaternary **T**→**R** transition simultaneously to a *full* **t**→**r** transition of the β subunits, or (b), a *full* **T**→**R** transition simultaneously to a *partial* **t**→**r** transition of the β subunits. Subsequently, the remaining quaternary transition (c) or the remaining tertiary transition of the β subunits was found to occur (d). The transitions of the two β subunits frequently occurred time-delayed to each other, rationalizing the low mutual information between the tertiary transitions of the two β subunits (Fig. 6B). Thus, an intermediate pathway between (a/c) and (b/d) is not excluded, with the transition of β1 being completed simultaneously to a partial quaternary transition (Fig. 5C), and the transition of the β2 subunit completed simultaneously to the remaining quaternary transition (Fig. 5D). The remaining **t**→**r** transition of the α subunits was typically not observed or occurred only partly during the simulations (e.g. Fig. 5A). In simulations starting from **R** (bottom of Fig. 8), the α subunits carried out a partial or full tertiary transition to **t** without a quaternary transition. Although we did not observe full **R**-**R2** transitions within simulation time, simulations starting from **R** and **R2** sample common configurational space (e.g., Fig. 2A/C and S2C), suggesting that **R** is in thermal equilibrium with **R2**.

## Materials and Methods

### Simulation setup

The initial structures for the simulations starting from the **R**, **R2**, and **T** state were taken from the protein data bank (PDB codes 1IRD [26], 1BBB [7] and 2HHB [27], respectively) and protonated using the pdb2gmx software. In simulations with deprotontated His(β)146, only the N_ε2_ atom of His146 was protonated. The **R**, **R2**, and **T** structures were placed into simulation boxes of a rhombic dodecahedron and solvated with 14057, 13980, and 14893 water molecules, respectively. 150mM of sodium chloride was added to each simulation box. Each box was neutralized by adding 10 more sodium ions (8 in T/R.noHC3 systems) than chloride ions. After energy minimization, each simulation system was simulated for 200ps with positions restraints on the backbone atoms to allow the relaxation of the water shell.

All simulations were carried out using the Gromacs simulation software [37], [38]. The GROMOS 43a2 force field [39] and the SPC [40] water model were applied. Electrostatic interactions were computed at every step using the particle-mesh Ewald algorithm [41], [42]. Short-range repulsive and attractive dispersion interactions were described by a Lennard-Jones potential, which was cut off at 1nm. The LINCS [43] and SETTLE [44] algorithms were used to constrain the protein and water bond lengths, respectively, allowing a time step of 2fs. Neighbor searching was carried out every 10 steps. The temperature was controlled at 300K through velocity rescaling (t = 2.5 ps) [45]. The pressure was kept constant by coupling the systems to a Parrinello-Rahman barostat of 1bar and τ = 5 ps [46].

In simulations with restrained salt bridges (T.BSres-1 to T.BSres-3), all salt bridges which are present in the **T** state but released in the **R** state were restrained by adding an additional harmonic potential (*k* = 2000 kJ/mol/nm^2^) between the respective pairs of atoms, with the potential equal to zero at the distance taken from the **T** X-ray structure. The restrained salt bridges are the inter-subunit salt bridges Val(α)1-R(α)141, D(α)126-R(α)141, K(α)40-H(β)146, as well as the intra-subunit salt bridge D(β)94-H(β)146, summing up to a total of 8 restrained salt bridges.

#### Mutual information as correlation measure

The correlations between the tertiary transitions of the α and β subunits and the quaternary transition of Hb were measured using the (Shannon) mutual information (MI) [47]. MI is based on information theory and captures all kinds of (equal-time) correlation, including non-linear and higher-order correlation. The MI *I*(*X*, *Y*) between two random variables *X* and *Y* is defined by
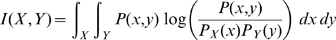
(1)Here, *P*(*x*, *y*) denotes the joint probability distribution of *X* and *Y*, and *P_X_*(*x*) and *P_Y_*(*y*) denote the marginal probability distributions of *X* and *Y*, respectively. Note that if (and only if) *X* and *Y* are independent, *P*(*x*, *y*) = *P_X_*(*x*)·*P_Y_*(*y*) holds, the logarithm in eq. 1 vanishes and the MI equals zero. Hence, the MI can be interpreted as the probability-weighted deviation of the case of *X* and *Y* being independent. The computation of the MI from the finite data sets is explained in detail in Text S1 and illustrated in Fig. S6.

## Supporting Information

Text S1 Computation of the mutual information
(0.05 MB DOC)

Figure S1 PCA projections of Hb simulations starting from the R X-ray structure with deprotonated His(β)146. (A–C) Projections of Hb structures during simulations R.noHC3-1 to R.noHC3-3 on the two eigenvectors derived from a PCA of the **T**, **R**, and **R2** X-ray structures. The color encodes the rotRMSD to **T**. (D) rotRMSD of simulations R.noHC3-1 to R.noHC3-3 to the **T** X-ray structure, confirming that the simulations do not approach **T**. (E) rotRMSD of R.noHC3-1 to R.noHC3-3 to the initial **R** structure during the first 50ns. The figure serves to choose a reasonable threshold of the rotRMSD to **R** that is henceforth applied on simulations starting from **T** to detect full **T**→**R** transitions. The **T**→**R** transitions were considered complete if the rotRMSD to **R** was smaller than 0.3nm, in the order of the rotRMSD of simulations R.noHC3 (E) after a few nanoseconds of equilibration (see section “Interplay between quaternary and tertiary conformational transitions”).
(0.83 MB TIF)

Figure S2 PCA projections of Hb simulations starting from the R2 X-ray structure. (A–C) Projections of Hb structures during simulations R2-1 to R2-3 on the two eigenvectors derived from a PCA of the **T**, **R**, and **R2** X-ray structures. The color encodes the rotRMSD to **R2**. (D) rotRMSD of simulations R2-1 to R2-3 to the R2 X-ray structure.
(0.76 MB TIF)

Figure S3 PCA projections of Hb simulations starting from the T X-ray structure with deprotonated His(β)146. (A–C, E–G) Projections of Hb structures during simulations T.noHC3-1 to T.noHC3-6 on the two eigenvectors derived from a PCA of the **T**, **R**, and **R2** X-ray structures. The color encodes the rotRMSD to the **R** X-ray structure. (D/H) rotRMSD of simulations T.noHC3-1 to T.noHC3-6 to the **R** X-ray structure. T.noHC3-1 carries out the full quaternary transition to **R**, whereas T.noHC3-4 and T.noHC3-6 carry out a partial transition to **R**.
(1.80 MB TIF)

Figure S4 rotRMSD of six of the 20 50ns-simulations starting from **T** state with deprotonated His(β)146. The six simulations shown here carried out the full **T**→**R** transition defined from a rotRMSD to the **R** X-ray structure of smaller 0.3nm, whereas the other 14 simulations did not approach the **R** state (not shown) and were not used for further analysis. Only simulation frames up to the closest approach to **R** (circles) were subsequently used to compute the mutual information. The red diamond indicates the rotRMSD of the **T** X-ray structure.
(0.27 MB TIF)

Figure S5 PCA projections of Hb simulations starting from the T X-ray structure with restrained salt bridges during the first 100ns (termed T.SBres-x). During the first 100ns of these simulations, all salt bridges that are present in the **T** structure but are released in the **R** structure were restrained by applying an additional harmonic potential between each pair of salt bridge partners (see Experimental procedures for detail). After 100ns, all salt bridge restraints were released. The simulations were carried out to exclude (i), that the tendency towards **R** is only an artifact from a possible underestimation of the salt bridge stability of the applied force field, and (ii), that the tendency towards **R** stems from insufficient equilibration of simulations starting at **T**. (A–C) Projections of Hb structures during simulations T.SBres-1 to T.SBres-3 on the two eigenvectors derived from a PCA of the **T**, **R**, and **R2** X-ray structures. Simulations frames during the first 100ns with restrained salt bridges are indicated by green dots, and simulation frames during the last 100ns without salt bridge restraints are indicated by magenta dots. (D) rotRMSD of simulations T.SBres-1 to T.SBres-3 to the **R** X-ray structure. Even with restrained salt bridges, Hb shows the same tendency towards **R** (green dots) as the simulations without restrained salt bridges (e.g., T.HC3-1-3), proving that the tendency towards **R** is an inherent property of Hb and not merely induced by a possible underestimation of the salt bridge stability. Note that the transitions to **R** are not complete during the first 100ns because the salt bridges would have to break upon a full transition to **R**. Among these simulations, T.SBres-3 carries out the full transition to **R** after releasing the salt bridge restraints (compare C and blue curve in D).
(0.84 MB TIF)

Figure S6 Computation of the mutual information (MI) from a finite number of pairs of data points. The MI was computed by extrapolating to an infinite number of data points, corresponding to k = 0. For details, see Text S1.
(0.07 MB TIF)

Figure S7 Projection on the quaternary difference vector between the **R** and **T** structure versus the rotation angle of the α2/β2 dimer during simulations (A) T.HC3-1-3 and (B) T.noHC3-1-6.
(0.28 MB TIF)

Video S1 Motions along the two eigenvectors derived from a principal component analysis on the T, R, and R2 structures.
(0.65 MB MOV)

Video S2 The quaternary T→R transition during simulation T.HC3-3. The Hb backbone is show in cartoon representation, the heme groups are shown as sticks, and the iron atoms as spheres. The β1 and β2 subunits are colored in red and purple, respectively, and the α1 and α2 subunits in green and lime, respectively.
(8.13 MB MOV)
