# Supplementary material for: Spontaneous Quaternary and Tertiary T-R Transitions of Human Hemoglobin in Molecular Dynamics Simulation
Source: PLoS Comput Biol. 2010 May 6;6(5):e1000774. doi: 10.1371/journal.pcbi.1000774 (PMC2865513; doi:10.1371/journal.pcbi.1000774)
Supplement: Text S1 — Computation of the mutual information (0.05 MB DOC) [file pcbi.1000774.s001.doc]

**Supporting Information for**

***Spontaneous quaternary and tertiary transitions of human hemoglobin A in molecular dynamics simulation***

Jochen S. Hub, Marcus B. Kubitzki, and Bert L. de Groot

***Text S1***

**Computation of the mutual information**

The mutual information (MI) was estimated from a discrete data set by a binning procedure. Accordingly, the probability distributions *P_X_*(*x*) and *P_Y_*(*y*) were approximated by counting occupancies *N_X_*(*i*) and *N_Y_*(*j*) of *X* and *Y* in bins *i*,*j* = 1,…,*N*_b_. Likewise, *P*(*x*, *y*) was approximated by a two-dimensional binning, yielding the two-dimensional occupancy *N*(*i, j*). *N*_b_=10 bins were used in the present study, although *N*_b_ was found to have only a minor influence on the results. The MI between *X* and *Y* was computed via

(1)

where *N_f_* denotes the number of simulation frames, and Δx and Δy denote the bin widths of *N_X_* and *N_Y_*, respectively.

The MI computed via eq. (1) depends on the number *N_f_* of data points. For each MI estimate, we therefore computed the MI as a function of *N_f_* and extrapolated to *N_f_* → ∞, as shown for one example in Figure S6. Let *M* denote the total number of *X*-*Y* pairs. Then, the MI was successively computed from *N_f_* = *M*/*k* data points, where *k* = 1,…,9, yielding *I_k_*(X,Y) as a function of *k* (Fig. S6, black dots). Subsequently, a parabola was fitted to the *k*-versus-*I_k_* pairs, allowing one to extrapolate to *k*=0 and hence, to *N_f_* → ∞ (Fig. S6, black dashed line). To verify that the procedure can indeed account for artifacts from the finite number of data points, we used random numbers to generate *M* artificial pairs of data points *X*_a_-*Y*_a_ that were distributed according to *P_X_* and *P_Y_*, but were completely independent. The corresponding *I_k_*(*X*_a_,*Y*_a_) increased approximately linearly with increasing *k* (Fig. S6, red dots). Extrapolation of *I_k_*(*X*_a_,*Y*_a_) to *k*=0 (corresponding to *N_f_* → ∞) always yielded an MI equal to zero, as expected for the independent sets X_a_ and Y_a_ (red dashed line). This result shows that the extrapolation to *N_f_* → ∞ can indeed remove artifacts from a finite number of data points, and that the MI derived for the original data set measures true correlation.
