# Supplementary figures and images for: Spontaneous Quaternary and Tertiary T-R Transitions of Human Hemoglobin in Molecular Dynamics Simulation
Source: PLoS Comput Biol. 2010 May 6;6(5):e1000774. doi: 10.1371/journal.pcbi.1000774 (PMC2865513; doi:10.1371/journal.pcbi.1000774)

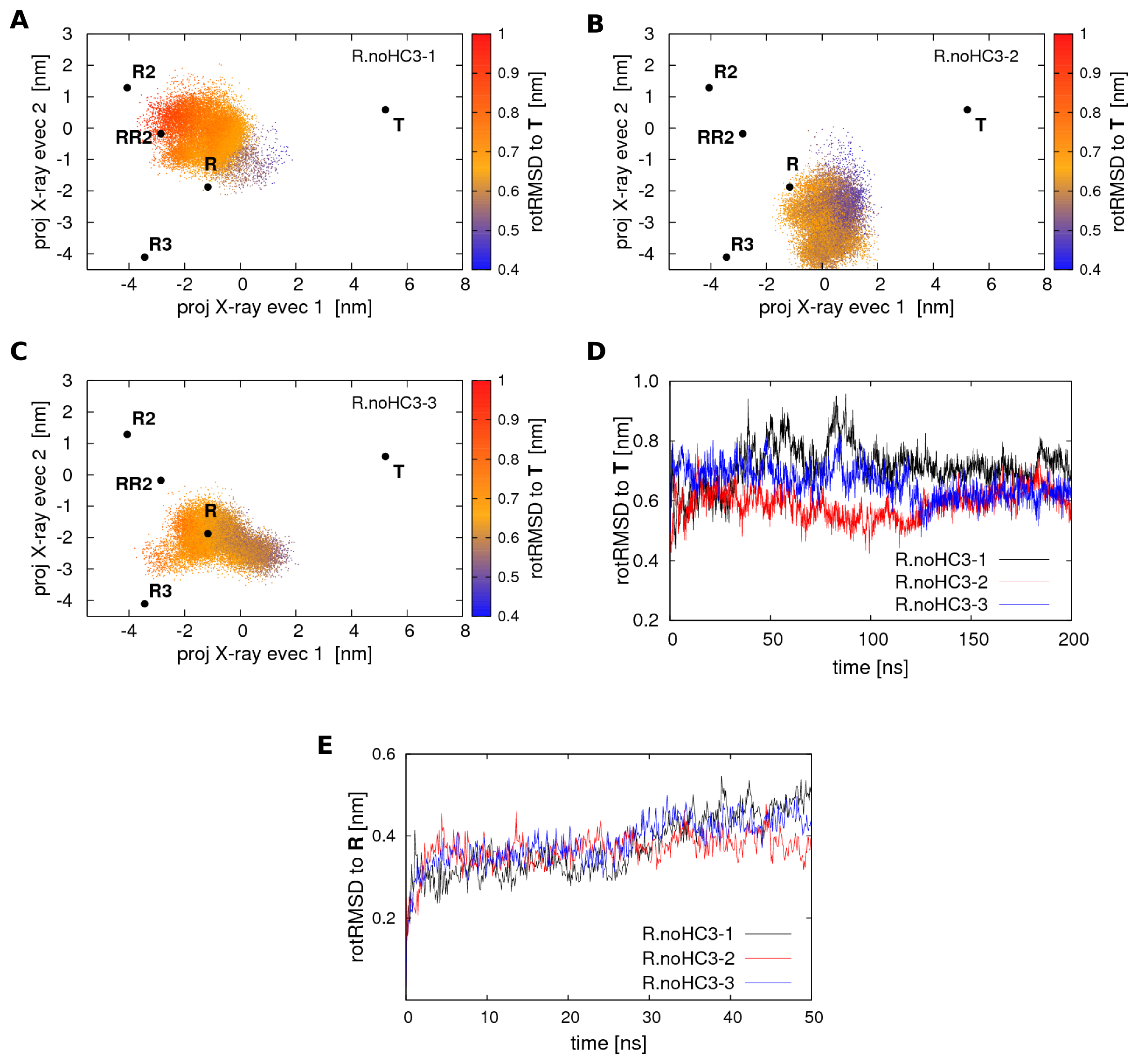

Supplement: Figure S1 — PCA projections of Hb simulations starting from the R X-ray structure with deprotonated His(β)146. (A–C) Projections of Hb structures during simulations R.noHC3-1 to R.noHC3-3 on the two eigenvectors derived from a PCA of the T, R, and R2 X-ray structures. The color encodes the rotRMSD to T. (D) rotRMSD of simulations R.noHC3-1 to R.noHC3-3 to the T X-ray structure, confirming that the simulations do not approach T. (E) rotRMSD of R.noHC3-1 to R.noHC3-3 to the initial R structure during the first 50ns. The figure serves to choose a reasonable threshold of the rotRMSD to R that is henceforth applied on simulations starting from T to detect full T→R transitions. The T→R transitions were considered complete if the rotRMSD to R was smaller than 0.3nm, in the order of the rotRMSD of simulations R.noHC3 (E) after a few nanoseconds of equilibration (see section “Interplay between quaternary and tertiary conformational transitions”). (0.83 MB TIF) [file pcbi.1000774.s002.tif]

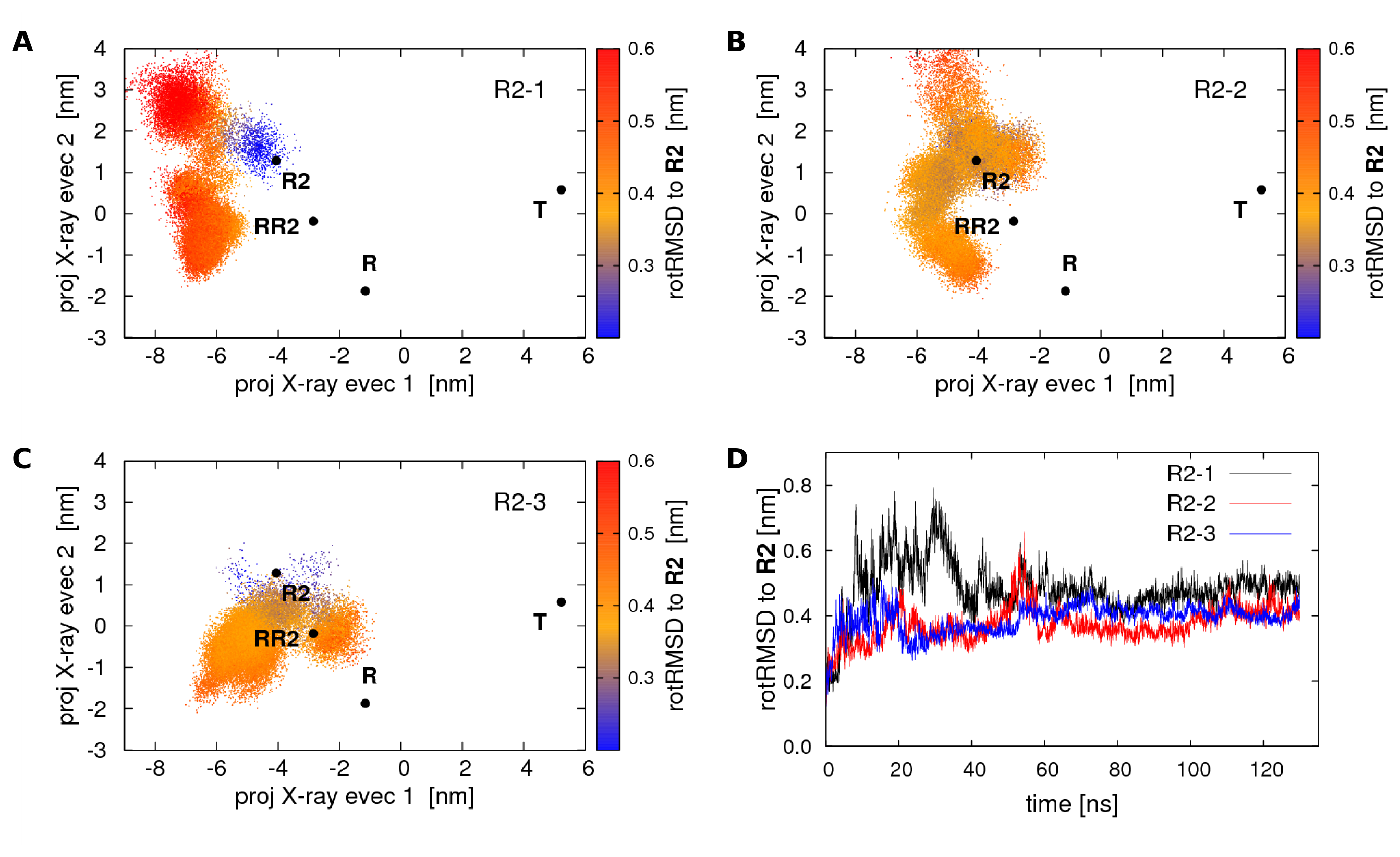

Supplement: Figure S2 — PCA projections of Hb simulations starting from the R2 X-ray structure. (A–C) Projections of Hb structures during simulations R2-1 to R2-3 on the two eigenvectors derived from a PCA of the T, R, and R2 X-ray structures. The color encodes the rotRMSD to R2. (D) rotRMSD of simulations R2-1 to R2-3 to the R2 X-ray structure. (0.76 MB TIF) [file pcbi.1000774.s003.tif]

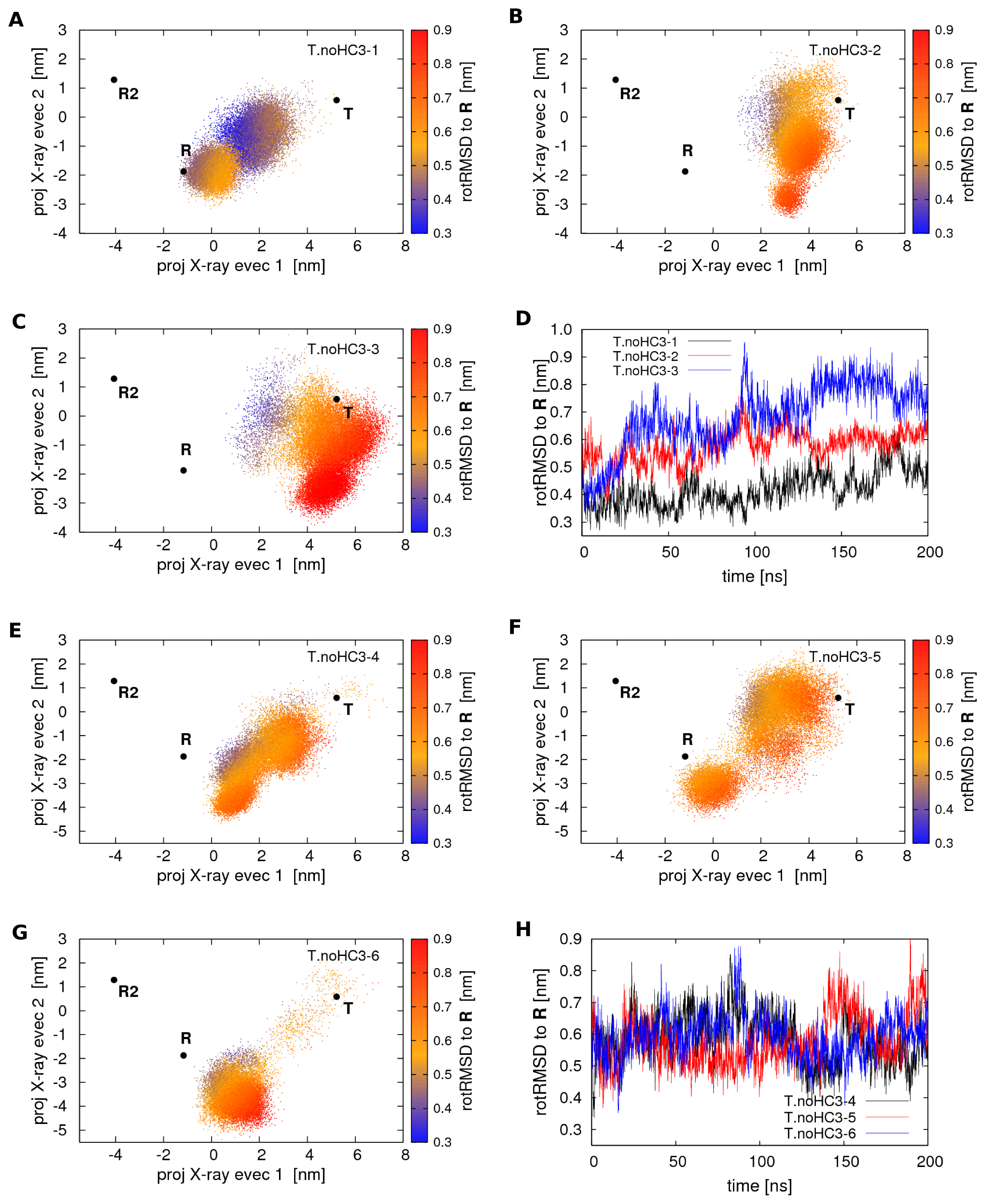

Supplement: Figure S3 — PCA projections of Hb simulations starting from the T X-ray structure with deprotonated His(β)146. (A–C, E–G) Projections of Hb structures during simulations T.noHC3-1 to T.noHC3-6 on the two eigenvectors derived from a PCA of the T, R, and R2 X-ray structures. The color encodes the rotRMSD to the R X-ray structure. (D/H) rotRMSD of simulations T.noHC3-1 to T.noHC3-6 to the R X-ray structure. T.noHC3-1 carries out the full quaternary transition to R, whereas T.noHC3-4 and T.noHC3-6 carry out a partial transition to R. (1.80 MB TIF) [file pcbi.1000774.s004.tif]

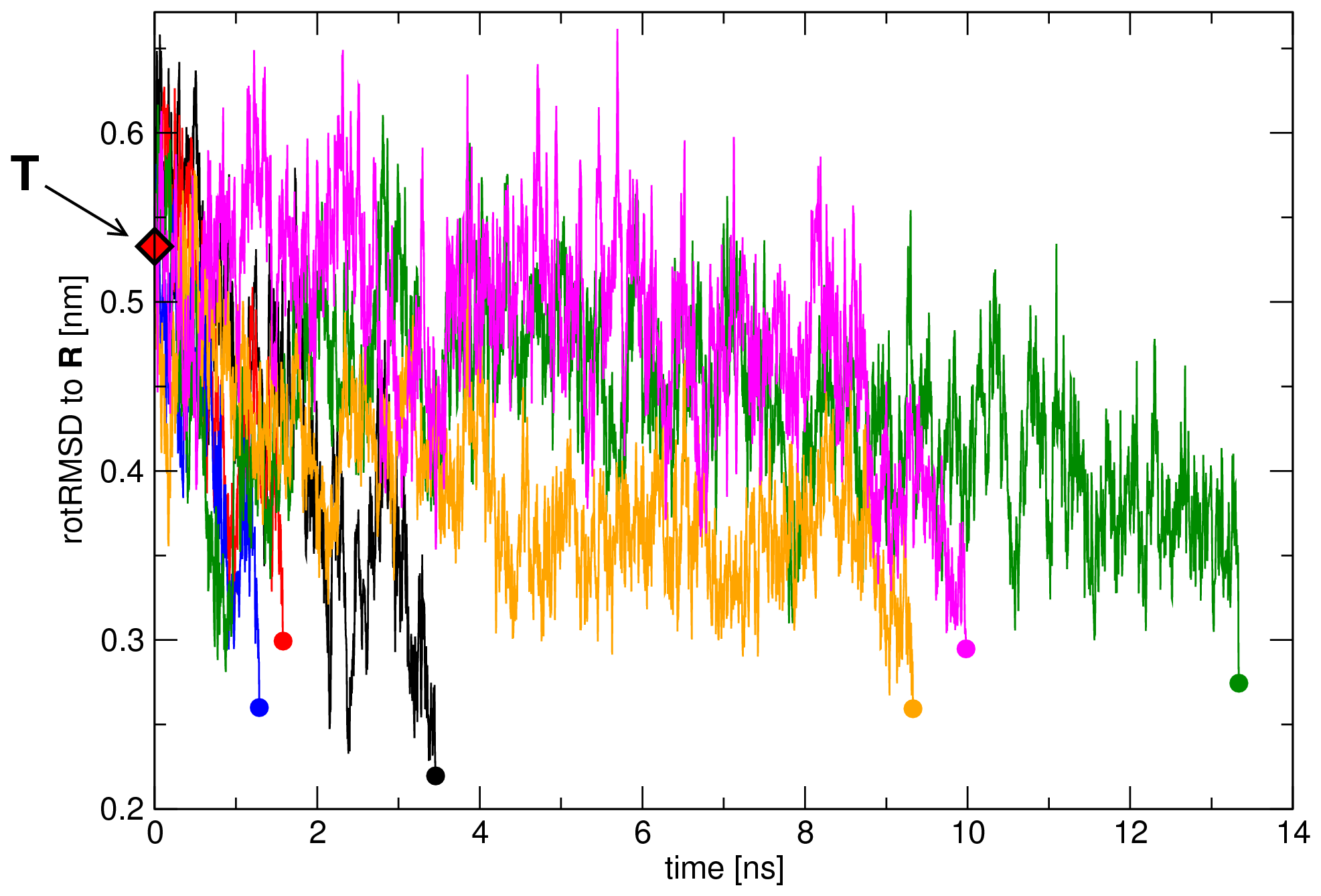

Supplement: Figure S4 — rotRMSD of six of the 20 50ns-simulations starting from T state with deprotonated His(β)146. The six simulations shown here carried out the full T→R transition defined from a rotRMSD to the R X-ray structure of smaller 0.3nm, whereas the other 14 simulations did not approach the R state (not shown) and were not used for further analysis. Only simulation frames up to the closest approach to R (circles) were subsequently used to compute the mutual information. The red diamond indicates the rotRMSD of the T X-ray structure. (0.27 MB TIF) [file pcbi.1000774.s005.tif]

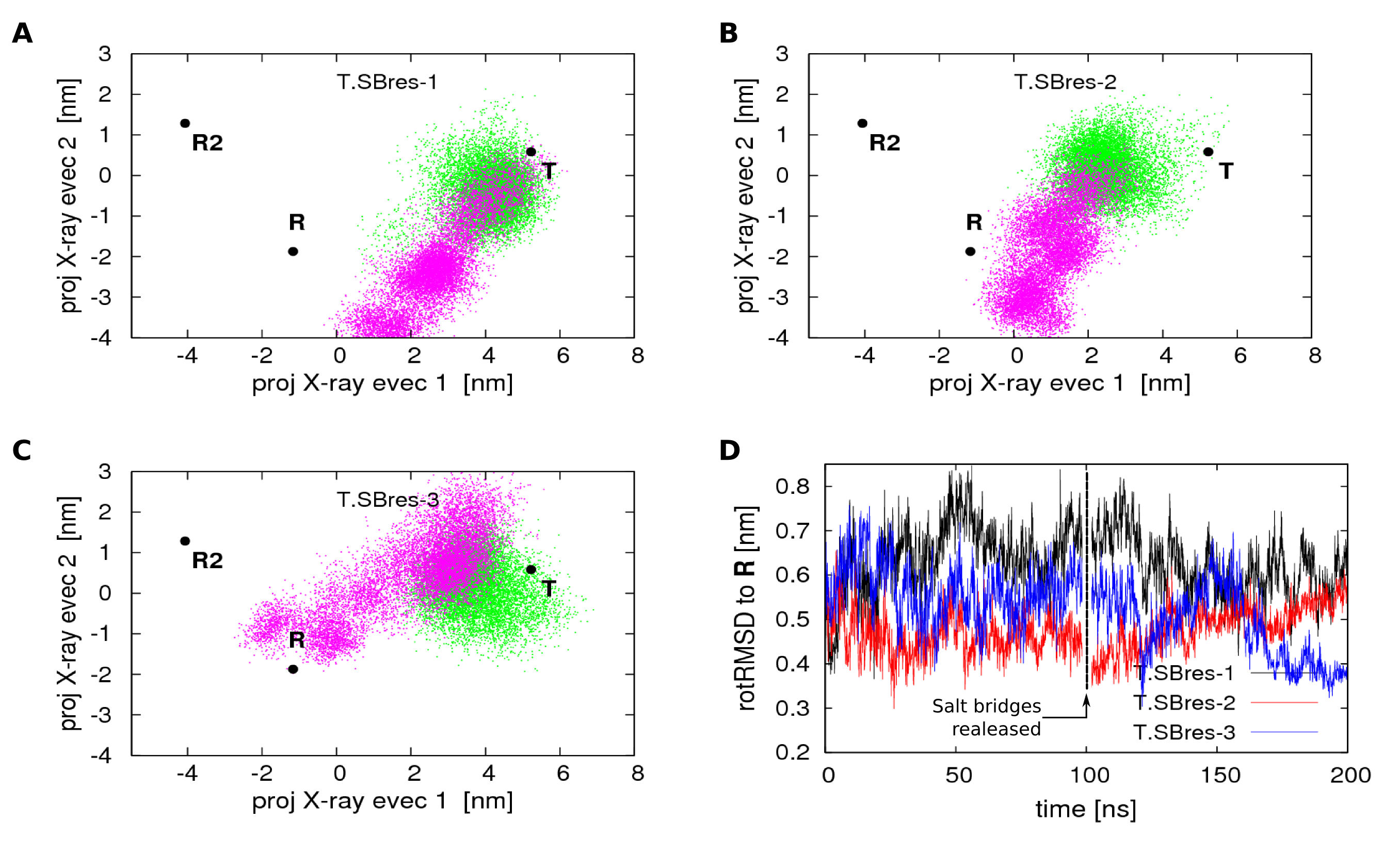

Supplement: Figure S5 — PCA projections of Hb simulations starting from the T X-ray structure with restrained salt bridges during the first 100ns (termed T.SBres-x). During the first 100ns of these simulations, all salt bridges that are present in the T structure but are released in the R structure were restrained by applying an additional harmonic potential between each pair of salt bridge partners (see Experimental procedures for detail). After 100ns, all salt bridge restraints were released. The simulations were carried out to exclude (i), that the tendency towards R is only an artifact from a possible underestimation of the salt bridge stability of the applied force field, and (ii), that the tendency towards R stems from insufficient equilibration of simulations starting at T. (A–C) Projections of Hb structures during simulations T.SBres-1 to T.SBres-3 on the two eigenvectors derived from a PCA of the T, R, and R2 X-ray structures. Simulations frames during the first 100ns with restrained salt bridges are indicated by green dots, and simulation frames during the last 100ns without salt bridge restraints are indicated by magenta dots. (D) rotRMSD of simulations T.SBres-1 to T.SBres-3 to the R X-ray structure. Even with restrained salt bridges, Hb shows the same tendency towards R (green dots) as the simulations without restrained salt bridges (e.g., T.HC3-1-3), proving that the tendency towards R is an inherent property of Hb and not merely induced by a possible underestimation of the salt bridge stability. Note that the transitions to R are not complete during the first 100ns because the salt bridges would have to break upon a full transition to R. Among these simulations, T.SBres-3 carries out the full transition to R after releasing the salt bridge restraints (compare C and blue curve in D). (0.84 MB TIF) [file pcbi.1000774.s006.tif]

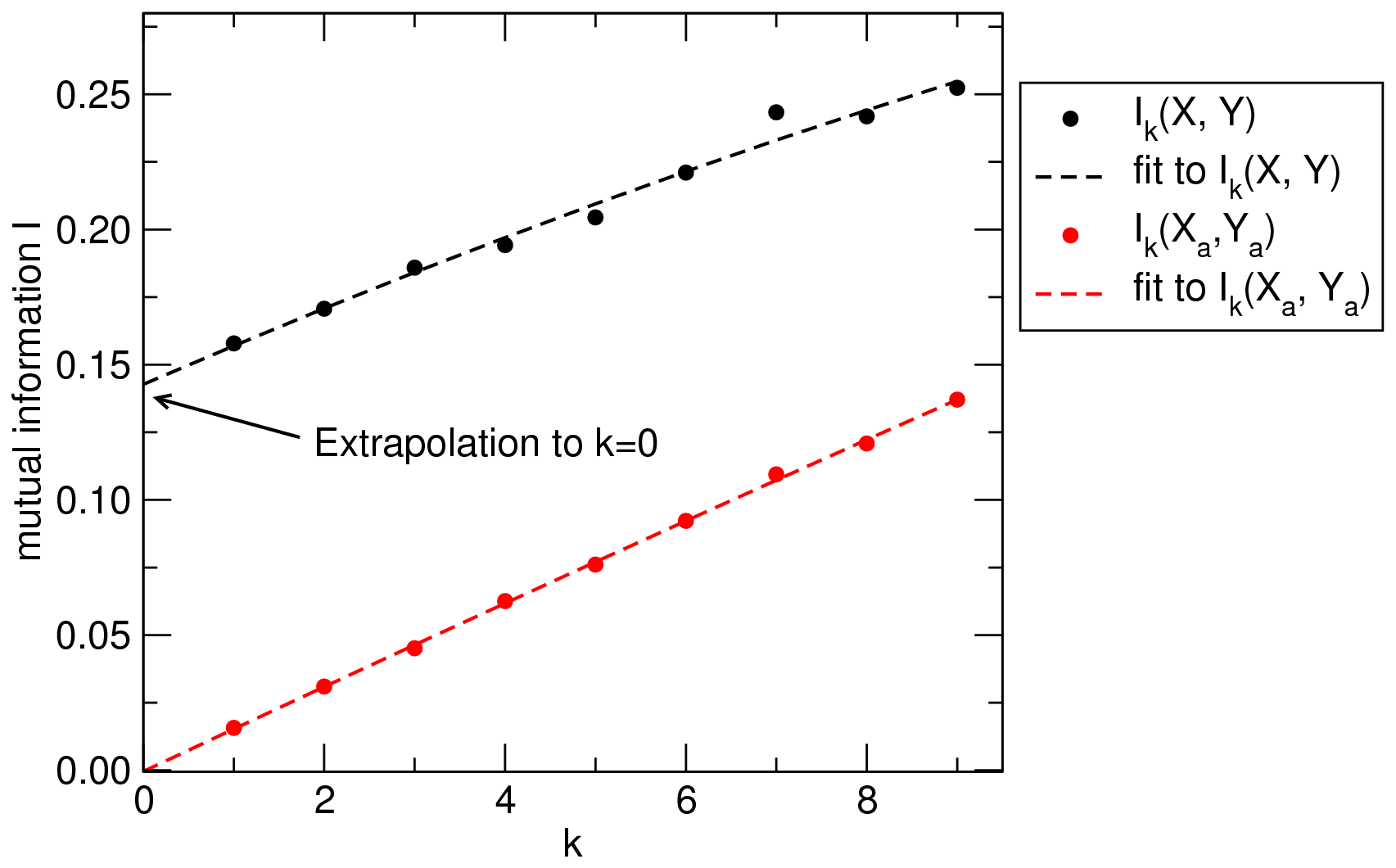

Supplement: Figure S6 — Computation of the mutual information (MI) from a finite number of pairs of data points. The MI was computed by extrapolating to an infinite number of data points, corresponding to k = 0. For details, see Text S1. (0.07 MB TIF) [file pcbi.1000774.s007.tif]

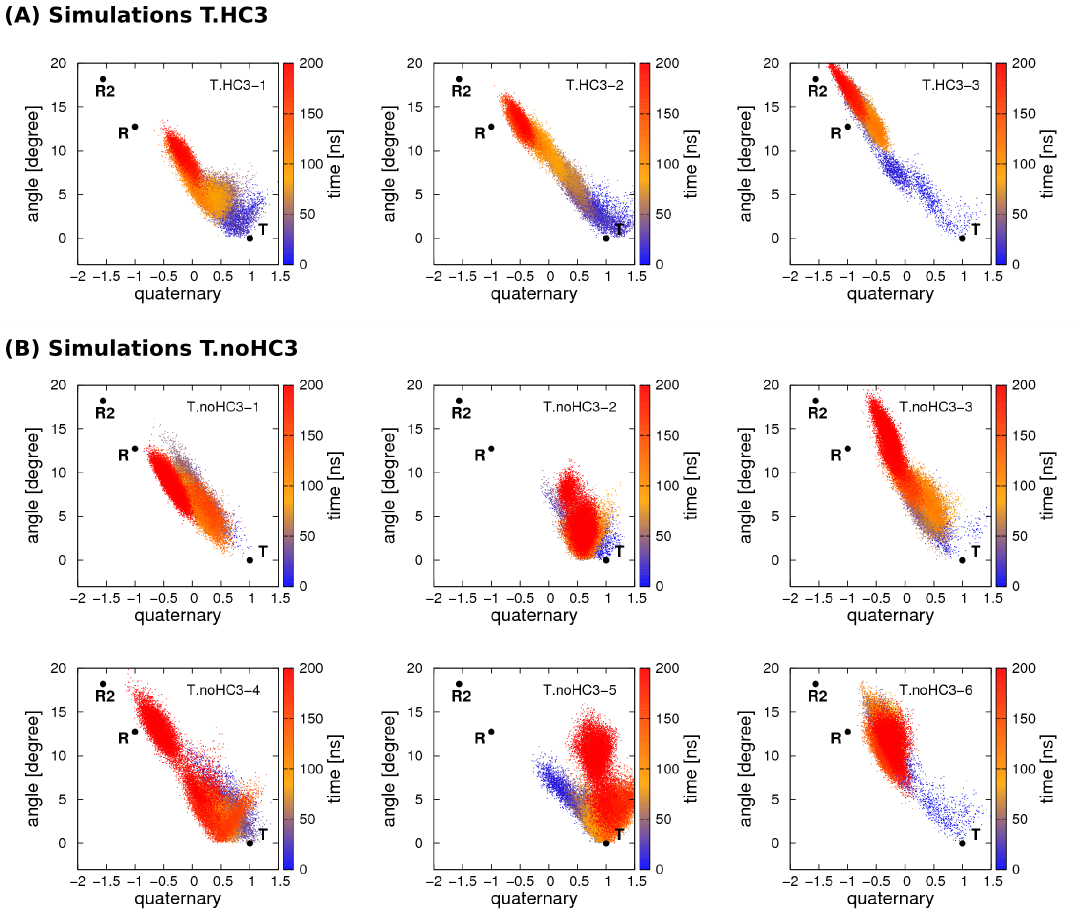

Supplement: Figure S7 — Projection on the quaternary difference vector between the R and T structure versus the rotation angle of the α2/β2 dimer during simulations (A) T.HC3-1-3 and (B) T.noHC3-1-6. (0.28 MB TIF) [file pcbi.1000774.s008.tif]
